# Synthesis and electrical property of metal/ZnO coaxial nanocables

**DOI:** 10.1186/1556-276X-7-316

**Published:** 2012-06-19

**Authors:** Zhi Li, Guanzhong Wang, Qianhui Yang, Zhibin Shao, Yang Wang

**Affiliations:** 1 Hefei National Laboratory for Physical Sciences at Microscale and Department of Physics, University of Science and Technology of China, Hefei, Anhui, 230026, People's Republic of China

**Keywords:** Ag, Cu, ZnO, Coaxial nanocables, Vapor-liquid-solid process, Schottky contact

## Abstract

Ag/ZnO and Cu/ZnO coaxial nanocables were fabricated using AgNO_3_ or copper foil as source materials by the vapor-liquid-solid process. The coaxial nanocables consist of a crystalline metallic Ag or Cu core and a semiconductor ZnO shell. The evolution of the Ag/ZnO products having different morphologies was investigated by stopping the heating at different temperatures. The diameters of the Ag/ZnO nanocables and the Ag cores could be modulated by changing Ag ratio in the source. The electrical characteristics of the Ag/ZnO contact and the influence of annealing reveal a Schottky diode behavior for a single Ag/ZnO nanocable device. The nanocables with uniform shape and controlled size are expected to provide a new choice in various applications of biological detection, nanothermometer, and photocatalysis.

## Background

Metal-semiconductor heterogeneous nanostructures have attracted particular attention due to their unique optical, electrical, and catalytic properties [1-3]. ZnO, with a direct wide bandgap energy of 3.37 eV at room temperature, is an important short-wavelength optoelectronic material and has drawn much attention. An enormous variety of ZnO nanostructures such as nanowires, nanobelts, and nanocables has been synthesized by a variety of techniques [4-6]. In particular, metal-ZnO nano-heterostructures have become an active frontier because of their wide application in dye-sensitized solar cells, photocatalysis, and biological detection [7-14]. For example, metal-ZnO Schottky diode is a fundamental component of a device for realizing one-dimensional (1D) nanoelectronics, which is useful for hydrogen sensor, strain sensor, and electrical switch [15-17]. Recently, some metal-ZnO nanostructures have been prepared by wet chemical routes, such as Au/ZnO hybrid nanoparticles, Pb/ZnO nanocables, and ZnO loaded with metal tips or dots [18-24]. However, nanostructures synthesized by wet chemical route usually have poor crystalline quality. The vapor-liquid-solid (VLS) method has been considered as the most promising method for fabricating 1D nanostructures, owing to the fact that the size of the nanostructures can be precisely controlled by the metal catalyst. A variety of functional 1D nanostructures, including silicon nanowires, carbon nanotubes, GaP nanowires, and ZnO nanobelts, has been demonstrated [25-28]. In addition, as far as we know, only Pb/ZnO and Zn/ZnO nanocables were studied in the case of metal/ZnO nanocables [20,29] Moreover, few properties of the metal-ZnO heterostructures were investigated, especially their electrical characteristics. Therefore, it is important to explore reliable approaches to synthesize metal/ZnO coaxial nanocables with different metal components and to study their contact characteristics.

Here, we report a one-step thermal evaporation route to fabricate the Ag/ZnO and Cu/ZnO coaxial nanocables via the VLS growth mechanism. The coaxial nanocables consist of a crystalline metallic Ag or Cu core and a semiconductor ZnO shell. The diameters of Ag/ZnO nanocables and the Ag cores could be modulated by changing Ag ratio in the source. The electrical characteristics of the Ag/ZnO contact and the influence of annealing on the contact were investigated. This approach can be extended to synthesize other metal/semiconductor coaxial nanocables for applications of the nanodevices.

## Methods

Ag/ZnO nanocables were synthesized in a conventional tube furnace using AgNO_3_ and ZnO as the source. An amount of 0.5 g of AgNO_3_ powder was placed at the bottom of an alumina boat, and 1 g of ZnO powder was added on the AgNO_3_ layer. The boat was put into the center of a ceramic tube that was mounted on the tube furnace. As a substrate, a Si (100) wafer was put in the low-temperature zone. The tube was evacuated to 10^−2^ Torr before it was heated, and the pressure was kept through the synthesizing process. No carrier gas was used in the whole process. It took about 35 min before the tube reached the desired temperature of about 1,300°C when the substrate temperature was about 950°C. Then, the heating was turned off, and the system was naturally cooled to room temperature. Cu/ZnO nanocables were obtained under the same condition except that 0.12 g copper foil, instead of AgNO_3_ powder, was used as the source material and that the substrate temperature was about 1,050°C.

The as-grown products were characterized by X-ray diffraction (XRD) with Cu Kα radiation (wavelength, 1.5045 Å) used as an incident X-ray source. Scanning electron microscopy (SEM) images were obtained on a field-emission SEM (JEOL JSM-6700 F, JEOL Ltd., Akishima, Tokyo, Japan). The products were also examined using a high-resolution transmission electron microscope (HRTEM, JEOL 2010, JEOL Ltd., Akishima, Tokyo, Japan) operating at 200 kV and X-ray photoelectron spectroscopy (XPS; VG ESCALAB X-ray photoelectron spectra spectrometer, VG Scientia Inc., Pleasanton, CA, USA). Their components were determined via energy-dispersive X-ray spectroscopy (EDS) attached in the HRTEM system. Photoluminescence (PL) spectra were measured at room temperature using a He-Cd laser (325 nm) as excitation source.

The electrical measurements of a single Ag/ZnO nanocable were performed by a one-axis manual linear translation stage. A nanocable was affixed to the tip of a tungsten probe attached on the stage by conductive silver epoxy. The nanocable was driven by the stage to approach an Au foil gradually until the Ag particle at the tip of the nanocable was in contact with the Au foil. The current-voltage (*I*-*V*) characteristics were measured by a picoammeter/voltage source (Keithley 6487 model, Keithley Instruments Inc., Cleveland, OH, USA) when voltage was applied between the ZnO shell and the Ag core.

## Results and discussion

**Figure 1:**
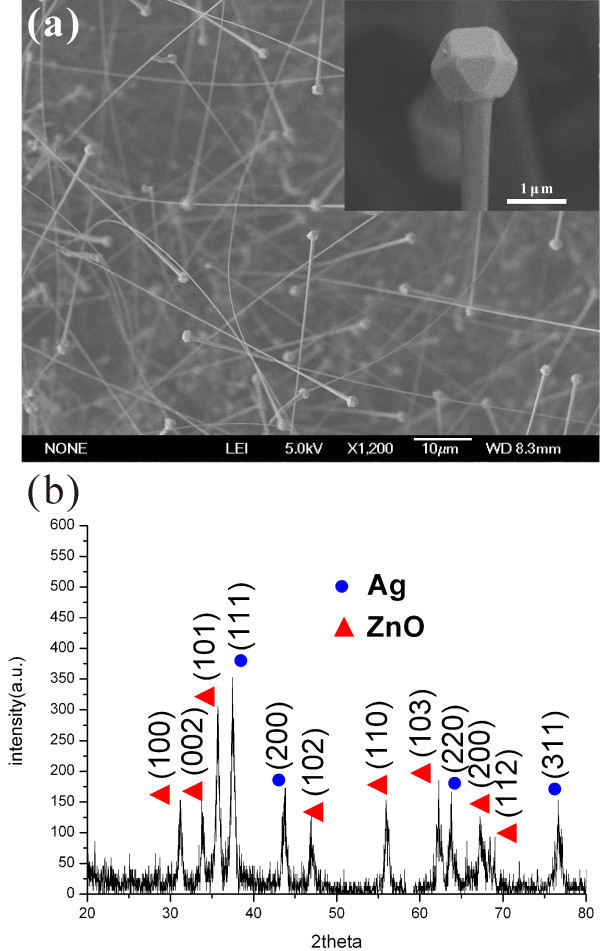
**SEM images and XRD pattern of the products.** (**a**) SEM image of the products obtained using ZnO and AgNO_3_ as the source. The inset is a higher magnification image. (**b**) A typical XRD pattern of the products.

(Figure 1a) shows a SEM image of the as-synthesized products using ZnO and AgNO_3_ as the source. Most of them are uniform nanowires with nanoparticles attached on the freestanding end. The lengths of the wires are up to 50 μm, and the diameters are in the range of 0.4 to 1.0 μm. The particles are truncated octahedrons as shown in the inset of (Figure 1a). The XRD pattern taken from this sample is given in (Figure 1b). All the peaks can be assigned to hexagonal ZnO and face-center-cubic Ag. No other peak is observed.

**Figure 2:**
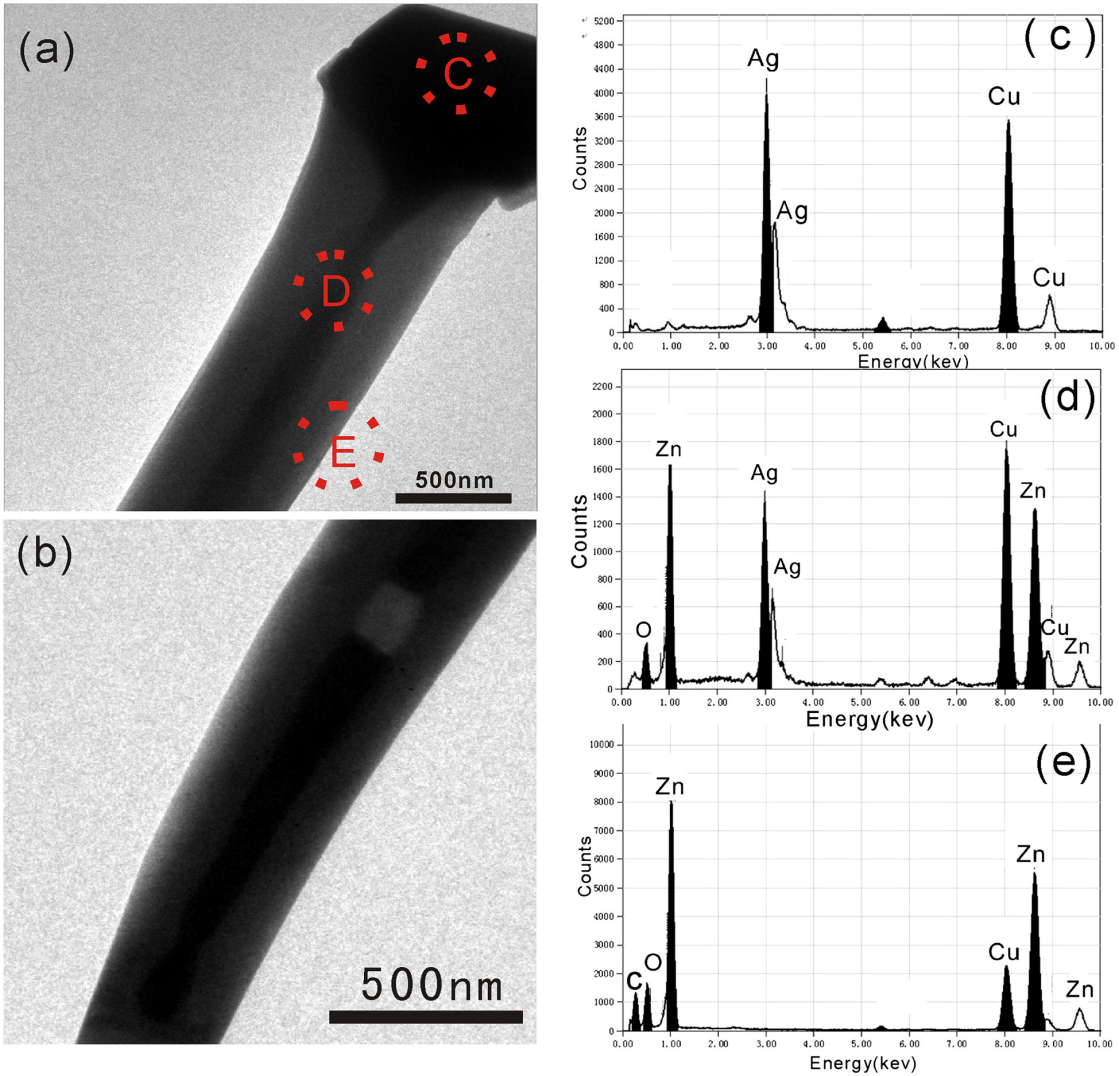
**TEM images and EDS spectra of an Ag/ZnO nanocable.** (**a**) The freestanding end with a Ag nanoparticle capped at the tip region. (**b**) The bottom end of the nanocable. (**c**,**d**,**e**) EDS spectra taken from the circled areas marked with C, D, and E in (a).

The detailed morphology and the chemical composition of the product were further characterized by transmission electron microscopy (TEM) and EDS, respectively. The TEM image of the tip region of a nanowire reveals that the nanowire has a coaxial nanocable structure with distinct interface as shown in (Figure 2a). It is clearly recognized that the polyhedron particle on top of the nanocable was connected with the inner core, which is an obvious evidence of the VLS growth mechanism. The same contrast in the TEM image of the connected core and the particle suggests that both the core and the particle are possibly the same material, which is confirmed by the EDS spectra. (Figure 2b) shows the TEM image of the other end of the same nanocable, which describes the portion where the core started. The inner core part was not always continuous across the whole nanocable, but a cavity could appear between two segments of the core in some nanocables. Most of the cavities in nanocables were completely filled according to our TEM observations, and the nanocables can be looked as filled nanotubes. To determine the compositions of the core and shell of the nanocable, EDS spectra were taken from different parts of the nanocable. (Figure 2c) shows the EDS spectrum taken from the capping particle (area C in Figure 2a), and only Ag and Cu signals are detected. The presence of Cu is likely due to the Cu TEM grid on which the sample was affixed. (Figure 2d) shows the EDS spectrum obtained from the middle of the nanocable (area D in Figure 2a). Besides Ag and Cu, Zn and O are also detected. The EDS spectrum taken from the shell of the nanocable (area E in Figure 2a) only shows the existence of Zn, O, and Cu. No Ag signal is detected. Taking all the observations into account, the nanocable is a heterostructure that a ZnO nanotube filled with metallic Ag except for a few unfilled segments in some cases.

**Figure 3:**
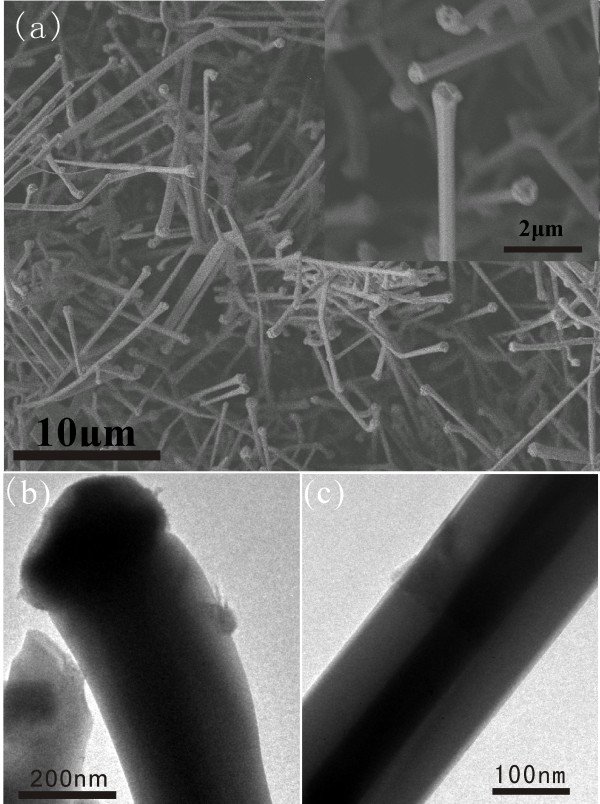
**SEM and TEM images of Cu/ZnO nanocables.** (**a**) SEM image of Cu/ZnO nanocables. The inset is a higher magnification image. (**b**,**c**) TEM images of Cu/ZnO nanocables.

The SEM image (Figure 3a) shows that the Cu/ZnO nanocables have an average diameter of 300 nm, smaller than the Ag/ZnO nanocables. The TEM images in (Figure 3b,c) and the corresponding EDS results (see Additional file 1) confirm that the nanocable contains a ZnO shell and a Cu core connected with a Cu particle at the tip. The growth of the Cu/ZnO nanocables was likely guided by the same mechanism of the growth of Ag/ZnO nanocables.

**Figure 4:**
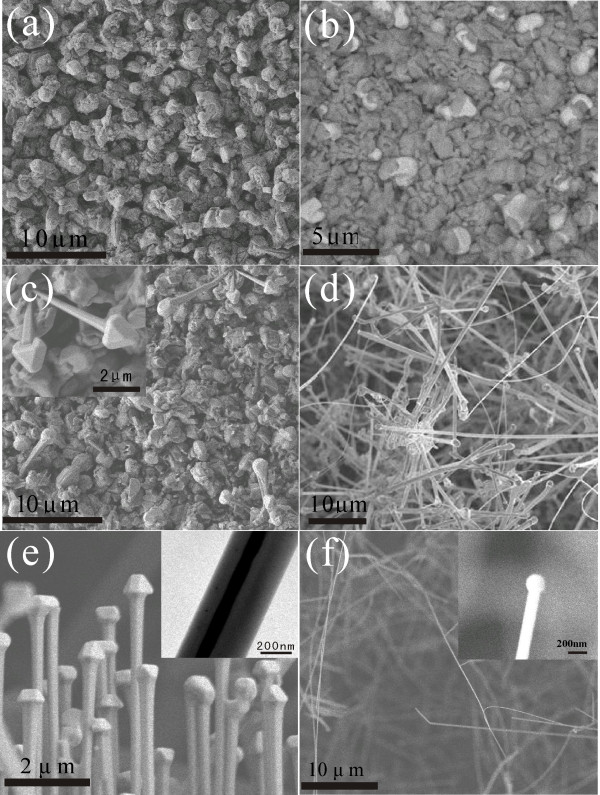
**Ag/ZnO nanocables synthesized under different conditions.** (**a**,**b**,**c**,**d**) SEM images of the products obtained using ZnO and AgNO_3_ as the source at reaction temperatures of 1,250°C, 1,280°C, 1,290°C, and 1,320°C, respectively. The inset in (c) is a higher magnification image. (**e**,**f**) SEM images of the products with AgNO_3_ of 0.25 and 0.1 g, respectively. The inset in (e) is a TEM image, and the inset in (f) is a higher magnification SEM image.

To understand the growth mechanism of these coaxial nanocables, synthesis of the Ag/ZnO nanocables under different conditions was conducted in an effort to observe the growth evolution of the nanocable structure. We kept the same heating power and stopped heating as soon as the temperature reached 1,250°C, 1,280°C, 1,290°C, and 1,320°C, respectively. The SEM images of the corresponding products are given in (Figure 4a,b,c,d), respectively. For the sample obtained from the heating stopped at 1,250°C or 1,280°C, only Ag-Zn alloy particles formed on the substrate (Figure 4a,b). If the heating was stopped when the temperature reached to 1,290°C, nanocables started to nucleate on the substrate (Figure 4c). When the temperature increased directly to 1,300°C after about 4 min of heating from 1,290°C, the nanocables could grow as long as 50 μm, as shown in (Figure 1a). Finally, high-density nanocables were grown as the temperature increased directly to 1,320°C (Figure 4d). (Figure 4e,f) shows the products fabricated with a reduced AgNO_3_ amount of 0.25 and 0.1 g while the amount of ZnO was kept the same, respectively. Products shown in (Figure 4e) still have a nanocable structure with a reduced diameter of 300 nm in average. Correspondingly, the diameter of the core decreased from 200 to 100 nm in average. When the amount of AgNO_3_ in the source was further decreased to 0.1 g, the diameter of the products decreased below 200 nm, as shown in (Figure 4f), and no evidence of Ag inner core was found in these products ( Figure S2c in Additional file 2). We suggest that the lower Ag concentration in the vapor results in smaller alloy particles formed at the initial growth stage, which changes the size of the wires. Smaller catalyst particle led to the decrease of the solid-liquid interface, and in this case, the center of the interface was easier to be oxidized to form ZnO if the oxygen concentration keeps the same. Thus, diameters of both the nanocable and the core decreased with reduction in the amount of AgNO_3_. No nanocables but ZnO nanowires were finally formed when the entire interface is oxidized [30]. Similar results were found for the Cu/ZnO nanocables. As shown in Additional file 2, SEM and TEM images reveal that only ZnO nanowires were formed as the substrate temperature decreased to 950°C.

**Figure 5:**
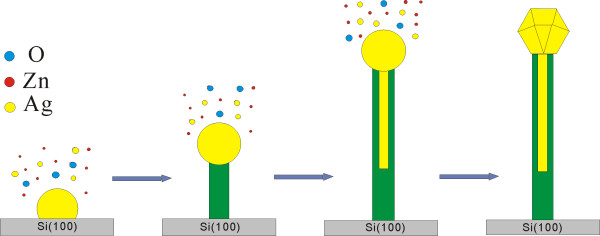
The schematic illustration of the growth models for Ag/ZnO nanocables.

A detailed schematic illustration of the formation process is shown in Figure 5. During the heating process, AgNO_3_ decomposed firstly due to its low decomposition temperature of 440°C. Because the AgNO_3_ was covered by ZnO powder in our experiments, Ag particles could be formed *in situ* from the decomposition of AgNO_3_. Then, with Ag vapor regenerated along with the increase of temperature, Ag droplets formed on the substrate and served as the catalyst. In the same time, Zn and O vapors were transported and adsorbed on the surface of the catalyst. When the alloy droplets became supersaturated, ZnO was phase-separated and crystallized to form ZnO nanowires as shown in (Figure 4c). With O content decreasing in the droplet, ZnO nanotubes started to form, and the liquid droplet was sucked into the hollow cavity due to the capillarity effect, similar to that observed in indium-filled In_2_O_3_ nanotubes [31]. Zn atoms diffused out from the alloy and were oxidized to ZnO, leaving Ag to fill the nanotube, same as shown in (Figure 2b). The droplets maintained their high activity to absorb Ag, Zn, and O vapors during the growth process, thereby resulting in continuous elongation of coaxial nanocables. As Zn and O were exhausted in the droplet after the reaction was finished, Ag was separated from ZnO during the slow cooling process [32]; a regular polyhedron shape of Ag crystal was finally formed at the tip because of the minimization of the surface energy [33]. Glaspell et al*.* have reported MgO and ZnO nanowires terminated by transition metal (Fe, Co, Ni) tips via the VLS process. The structures were similar to our results except having no inner core. They mentioned that the morphology of the tips depended on the nature of the metal catalyst. This can also explain the morphology difference between Ag and Cu nanoparticles capped at the tip region in our results [34]. Cu/ZnO nanocables were also successfully synthesized via a similar mechanism as that of Ag/ZnO, owing to the similarity between the Ag-Zn and Cu-Zn binary phase diagrams. This implies that the proposed method may be universal in preparing metal/semiconductor coaxial nanocables.

**Figure 6:**
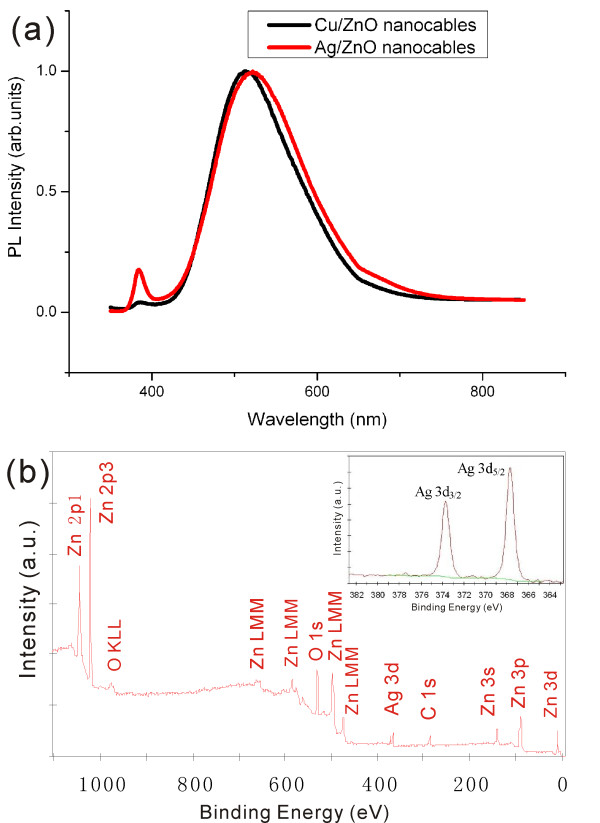
**PL spectra and XPS spectrum of the nanocables.** (**a**) PL spectra of Ag/ZnO and Cu/ZnO nanocables on Si substrate. Each spectrum is normalized by its maximal intensity for easy comparison. (**b**) The XPS spectrum of Ag/ZnO nanocables. The high-resolution spectra for Ag species are shown in the inset.

**Figure 7:**
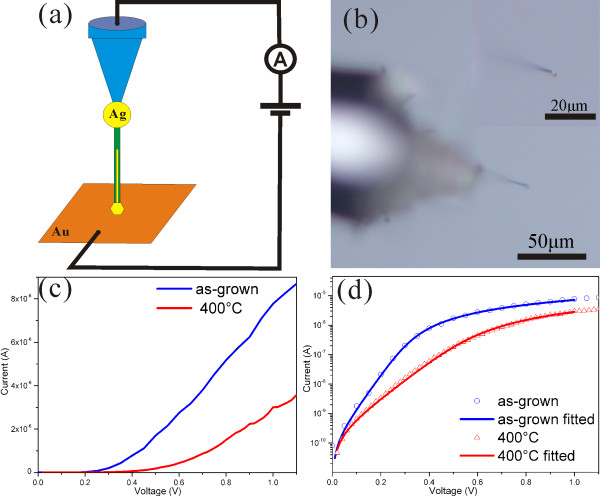
**The electrical characteristics of the Ag/ZnO contact and the influence of annealing.** (**a**) Schematic of the measurement system to characterize the performance of the device. (**b**) Optical image of the nanocable affixed to the probe. The inset is a higher magnification image of the tip region. (**c**) *I*-*V* characteristics of a single Ag/ZnO nanocable before and after annealing in the air at 400°C for 1 h. (**d**) Logarithm plot of the current using the data in (c).

The optical properties of the Ag/ZnO and Cu/ZnO nanocables were characterized by PL measurements at room temperature. As shown in (Figure 6a), the two characteristic emissions were detected for each sample. The band edge emission is centered at 384 nm for Ag/ZnO nanocables and 385 nm for Cu/ZnO nanocables. The deep trap emission due to defect states is around 522 nm for Ag/ZnO nanocables and 513 nm for Cu/ZnO nanocables. The deep trap emission is much more intense than the band edge emission for both samples compared to normal ZnO nanowires, indicating that the as-prepared nanostructures contained many defects related to oxygen vacancies [35]. The XPS analysis was carried out to investigate the surface structure of the Ag/ZnO nanocables. All peaks in the XPS full spectrum shown in (Figure 6b) can be ascribed to Zn, Ag, O, and C elements, which is consistent with the above XRD and EDS results. The high-resolution spectra for Ag species are shown in the inset in (Figure 6b). The binding energies of Ag 3*d*5/2 and Ag 3*d*3/2 for the Ag/ZnO nanocables are 367.7 and 373.7 eV, respectively, which shift remarkably to the lower binding energy compared with the corresponding values of the synthesized pure metallic Ag (the standard binding energies of Ag 3*d*5/2 and Ag 3*d*3/2 for bulk Ag are about 368.2 and 374.2 eV, respectively). Since the work function of silver (4.26 eV) is smaller than that of ZnO (5.2 to 5.3 eV), electrons will transfer from Ag to ZnO at the interfaces of the Ag/ZnO nanocables, resulting in a new Fermi energy level in Ag/ZnO nanocables. Therefore, the binding energies of Ag 3*d*5/2 and Ag 3*d*3/2 shift to the lower ones in the Ag/ZnO nanocables [13].

(Figure 7a,b) shows the schematic of the setup to measure the electrical properties of the Ag/ZnO contacts and the optical image of the Ag/ZnO nanocable affixed to the probe. Forward *I*-*V* characteristics from as-grown Ag/ZnO nanocables and those annealed at 400°C in air for 1 h are shown in (Figure 7c,d). The *I*-*V* characteristics exhibit well-defined rectifying behavior of a Schottky barrier diode structure, which mainly reflects current transport across the metal-semiconductor interface. It is described by the thermionic emission relation:

(1) $$I=SA^{*}\exp\left(-\frac{q\Phi_{\mathrm{SB}}}{k_{B}T}\right)\left\{\exp\left(\frac{qV-IR_{s}}{nk_{B}T}\right)-1\right\}\text{,}$$

where *I* is the current, *S* is the area of the Schottky barrier, A* is Richardson's constant for *n*-type ZnO, *Φ*_**SB**_ is the Schottky barrier height, *T* is the temperature, *V* is the applied voltage, *R*_**S**_ is the series resistance, and *n* is the ideality factor. Here, the *S* value is estimated by the diameter and length of the Ag core of 200 nm and 20 μm, respectively.

Table 1 shows a summary of the contact properties of the as-grown and annealed nanocables. The barrier height of the as-grown samples is 0.313 eV. The ideality factor *n* is a quantity for describing the deviation of the diode from an ideal Schottky barrier for which *n* = 1. The ideality factor of the Ag/ZnO Schottky barrier before annealing is determined to be 1.455, which is close to 1. *I*_**S**_ is the saturation current. The effect of the annealing is to lower the barrier height (from 0.313 eV in the as-grown samples to 0.298 eV in those annealed at 400°C) and increase the saturation current (from 1.004 × 10^−10^ A in the as-grown samples to 1.811 × 10^−10^ A in the annealed samples), which is consistent with the early report [36]. The changes can be attributed to the interface roughening after annealing.

**Table 1:** Schottky barrier properties of Ag-ZnO contact before and after 400°C annealing

**Sample**	***Φ***_**SB**_**(eV)**	***n***	***I***_**S**_**(A)**	***R***_**S**_**(Ω)**
As-grown Ag/ZnO nanocables	0.313	1.455	1.004 × 10^−10^	8.051 × 10^4^
Ag/ZnO nanocable after 400°C annealing in air	0.298	2.686	1.811 × 10^−10^	1.164 × 10^5^

## Conclusion

In summary, Ag/ZnO and Cu/ZnO coaxial nanocables were fabricated by thermally evaporating the source material of ZnO and AgNO_3_ (or copper foil) using the vapor-liquid-solid mechanism. SEM, TEM, and EDS results reveal that the coaxial nanocables consist of a crystalline metallic Ag or Cu core and a semiconductor ZnO shell. The diameters of Ag/ZnO nanocables and the Ag cores could be modulated by changing Ag ratio in the source. PL measurements show that Ag/ZnO and Cu/ZnO coaxial nanocables have the band edge emissions and the deep trap emissions due to defect states. The electrical characteristics of the Ag/ZnO contact and the influence of annealing reveal a Schottky diode behavior for a single Ag/ZnO nanocable device.

## Competing interests

The authors declare that they have no competing interests.

## Authors’ contributions

GW led the project and participated in the design of the experiments, analysis of the data, and revision of the manuscript. ZL designed and carried out the experiments and drafted the manuscript. ZS participated in the design. QY and YW contributed to the electrical testing. All authors read and approved the final manuscript.

## Supplementary Material

Additional file 1: **Figure S1.** Description: For Figure S1, (a to c) EDS spectra taken from the capping particle, the middle, and the shell of a Cu/ZnO nanocable from the sample shown in (Figure 3), respectively. Nickel grids were used in the measurement to make sure that the Cu signals come from the sample, not from the TEM grid. Click here for file

Additional file 2: **Figure S2.** Description: For Figure S2, (a) SEM and (b) TEM images of Cu/ZnO products obtained at the same experimental condition as the sample in (Figure 3 )except only the substrate is located in the low-temperature zone of 950°C. (c) TEM image of Ag/ZnO products obtained at the same experimental condition as the sample in (Figure 1) except only 0.1 g AgNO_3_ and 1 g ZnO were used as source materials. Click here for file
