# Supplementary material for: Synthesis and electrical property of metal/ZnO coaxial nanocables
Source: Nanoscale Res Lett. 2012 Jun 19;7(1):316. doi: 10.1186/1556-276X-7-316 (PMC3432616; doi:10.1186/1556-276X-7-316)
Supplement: Additional file 1: — Figure S1. Description: For Figure S1, (a to c) EDS spectra taken from the capping particle, the middle, and the shell of a Cu/ZnO nanocable from the sample shown in (Figure 3), respectively. Nickel grids were used in the measurement to make sure that the Cu signals come from the sample, not from the TEM grid. [file 1556-276X-7-316-S1.pdf]

Additional file 1

Synthesis and Electrical Property of Metal/ZnO Coaxial Nanocables

Zhi Li, Guanzhong Wang* , Qianhui Yang, Zhibin Shao, and Yang Wang

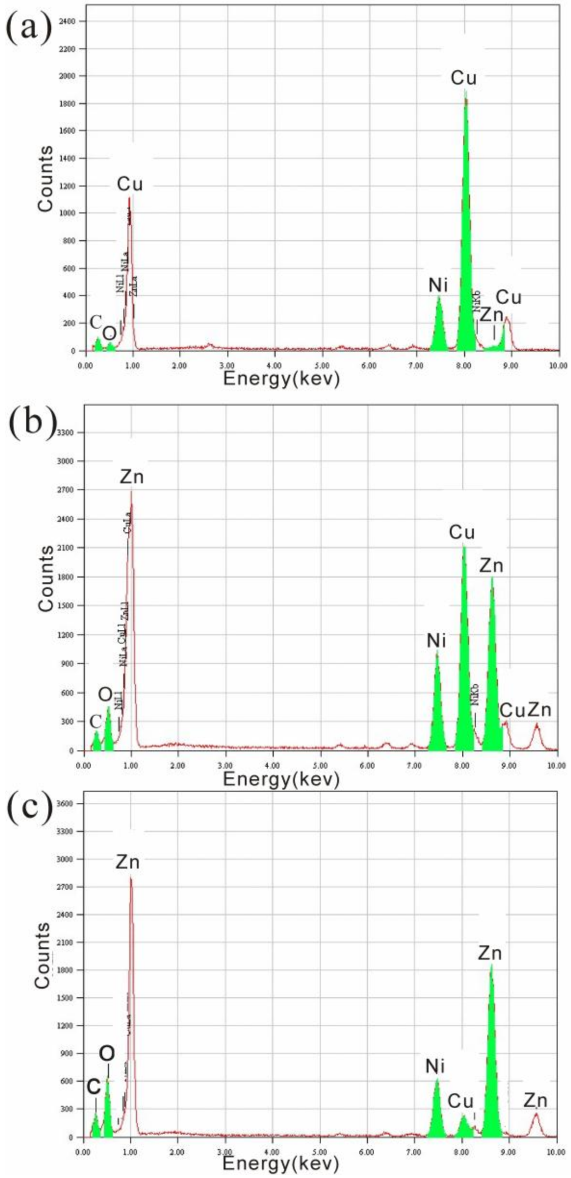

Figure S1. (a–c) EDS spectra taken from the capping particle, the middle and the shell of a Cu/ZnO nanocable from the sample shown in Figure 3, respectively. Nickel grids were used in the measurement to make sure that the Cu signals come from the sample not from the TEM grid.
