# Supplementary material for: Synthesis and electrical property of metal/ZnO coaxial nanocables
Source: Nanoscale Res Lett. 2012 Jun 19;7(1):316. doi: 10.1186/1556-276X-7-316 (PMC3432616; doi:10.1186/1556-276X-7-316)
Supplement: Additional file 2: — Figure S2. Description: For Figure S2, (a) SEM and (b) TEM images of Cu/ZnO products obtained at the same experimental condition as the sample in (Figure 3 )except only the substrate is located in the low-temperature zone of 950°C. (c) TEM image of Ag/ZnO products obtained at the same experimental condition as the sample in (Figure 1) except only 0.1 g AgNO3 and 1 g ZnO were used as source materials. [file 1556-276X-7-316-S2.pdf]

Additional file 2

Synthesis and Electrical Property of Metal/ZnO Coaxial Nanocables

Zhi Li, Guanzhong Wang* , Qianhui Yang, Zhibin Shao, and Yang Wang

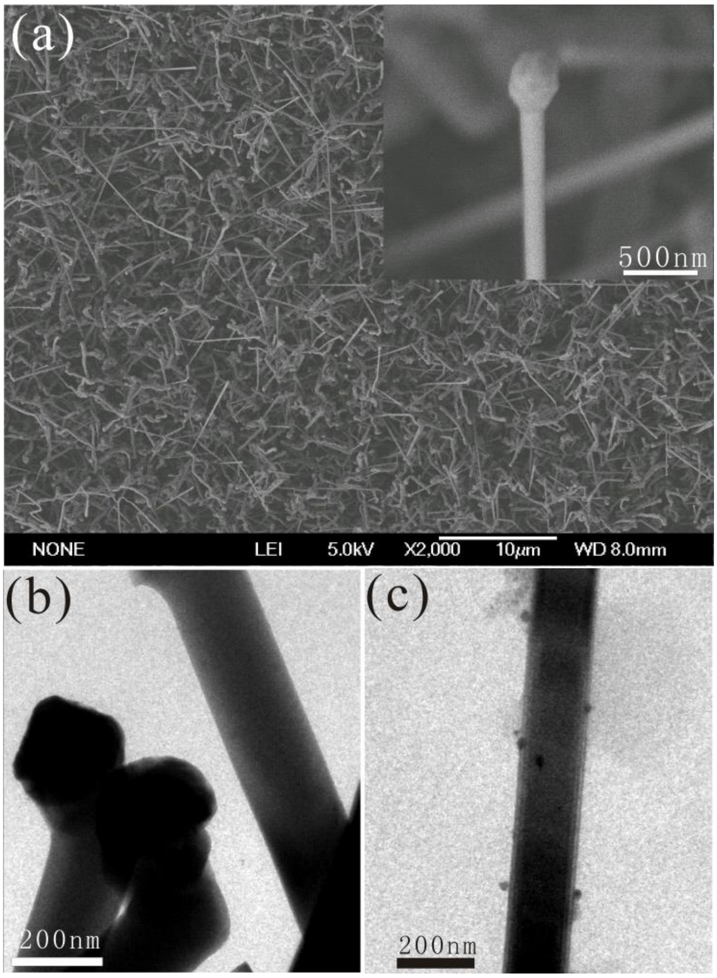

Figure S2. (a) SEM and (b) TEM images of Cu/ZnO products obtained at the same experimental condition as the sample in Figure 3 except only the substrate located in the low-temperature zone of 950 °C. (c) TEM image of Ag/ZnO products obtained at the same experimental condition as the sample in Figure 1 except only 0.1 g $AgNO_3$ and 1g ZnO as source material.
